# Epilation for Minor Trachomatous Trichiasis: Four-Year Results of a Randomised Controlled Trial

**DOI:** 10.1371/journal.pntd.0003558

**Published:** 2015-03-13

**Authors:** Esmael Habtamu, Saul N. Rajak, Zerihun Tadesse, Tariku Wondie, Mulat Zerihun, Birhan Guadie, Teshome Gebre, Amir Bedri Kello, Kelly Callahan, David C. W. Mabey, Peng T. Khaw, Clare E. Gilbert, Helen A. Weiss, Paul M. Emerson, Matthew J. Burton

**Affiliations:** 1 Faculty of Infectious and Tropical Diseases, London School of Hygiene & Tropical Medicine, London, United Kingdom; 2 The Carter Center, Addis Ababa, Ethiopia; 3 Amhara Regional Health Bureau, Bahirdar, Ethiopia; 4 International Trachoma Initiative, Addis Ababa, Ethiopia; 5 Light for the World, Addis Ababa, Ethiopia; 6 The Carter Center, Atlanta, Georgia, United States of America; 7 NIHR Biomedical Research Centre at Moorfields Eye Hospital and UCL Institute of Ophthalmology, London, United Kingdom; 8 MRC Tropical Epidemiology Group, London School of Hygiene & Tropical Medicine, London, United Kingdom; 9 International Trachoma Initiative, Atlanta, Georgia, United States of America; University of Cambridge, UNITED KINGDOM

## Abstract

**Background:**

Trachomatous trichiasis (TT) needs to be managed to reduce the risk of vision loss. The long-term impact of epilation (a common traditional practice of repeated plucking of lashes touching the eye) in preventing visual impairment and corneal opacity from TT is unknown. We conducted a randomized controlled trial of epilation versus surgery for the management of minor TT (fewer than six lashes touching the eye) in Ethiopia. Here we report the four-year outcome and the effect on vision and corneal opacity.

**Methodology/ Principal Findings:**

1300 individuals with minor TT were recruited and randomly assigned to quality trichiasis surgery or repeated epilation using high quality epilation forceps by a trained person with good near vision. Participants were examined six-monthly for two-years, and then at four-years after randomisation. At two-years all epilation arm participants were offered free surgery. At four-years 1151 (88.5%) were re-examined: 572 (88%) and 579 (89%) from epilation and surgery arms, respectively. At that time, 21.1% of the surgery arm participants had recurrent TT; 189/572 (33%) of the epilation arm had received surgery, while 383 (67%) declined surgery and had continued epilating (“epilation-only”). Among the epilation-only group, 207 (54.1%) fully controlled their TT, 166 (43.3%) had minor TT and 10 (2.6%) had major TT (&gt;5 lashes). There were no differences between participants in the epilation-only, epilation-to-surgery and surgery arm participants in changes in visual acuity and corneal opacity between baseline and four-years.

**Conclusions/ Significance:**

Most minor TT participants randomised to the epilation arm continued epilating and controlled their TT. Change in vision and corneal opacity was comparable between surgery and epilation-only participants. This suggests that good quality epilation with regular follow-up is a reasonable second-line alternative to surgery for minor TT for individuals who either decline surgery or do not have immediate access to surgical treatment.

## Author Summary

Trachoma causes visual impairment through the effect of in-turned eyelashes (trichiasis) on the surface of the eye. Epilation is a common traditional practice of intermittent plucking of lashes touching the eye, however, its long-term effectiveness in preventing visual impairment is unknown. We conducted a randomized controlled trial of epilation versus eyelid surgery (the main treatment option) in 1300 people with mild trichiasis in Ethiopia. We defined mild trichiasis as fewer than six lashes touching the eye. We have previously reported results to two years and have now re-assessed these individuals at four years. Overall, we found no difference between the epilation and surgery groups in terms of change in vision and corneal opacity between baseline and four years. Most mild trichiasis participants randomised to the epilation arm continued epilating and controlled their trichiasis. This suggests that good quality epilation is a reasonable second-line alternative to surgery for mild trichiasis for individuals who either decline surgery or do not have immediate access to surgical treatment.

## Introduction

Trachoma is the leading infectious cause of blindness worldwide [1]. Trachomatous trichiasis (TT) is the late stage scarring sequelae of repeated conjunctival *Chlamydia trachomatis* infection and inflammation in which the upper eyelid is distorted and rolled inwards (entropion) and the eyelashes turn towards the eye [2]. Trachoma leads to visual impairment through the damaging effect of trichiasis on the cornea. The risk of sight loss is directly correlated with disease severity, becoming more frequent with increasing severity of trichiasis [3–6]. The clinical phenotype ranges from a single aberrant eyelash touching the eye (without entropion) to the whole eyelid rolled inwards [7]. Some lashes may scratch the cornea directly while others are peripheral. Trichiasis is usually grouped, based on the number of eyelashes touching the eye into minor TT (1–5 lashes touching the eye) and major TT (>5 lashes touching the eye) [5,8]. Globally, the most recent World Health Organisation (WHO) estimate suggested 8 million people had trichiasis in 2009 [1]. Updated disease estimates will become available in the next few years from the Global Trachoma Mapping Programme.

Eyelid surgery is performed to correct the anatomical abnormality, in the expectation that this reduces the risk of sight loss [4,9]. The WHO advises that “all patients should be offered surgery for entropion trichiasis” [9]. However, up to half of the individuals with trachomatous trichiasis may not have significant entropion [7]. Therefore, there is a degree of ambiguity about how programmes should manage patients with non-entropic trichiasis, particularly those with only a few lashes touching the eye.

Despite considerable efforts to scale-up surgery programmes only around 150,000 people per year have been reported as treated surgically in recent years worldwide [10]. It has been estimated at the current rate the trichiasis backlog (ignoring incident cases) would not be dealt with until 2032, twelve years after the 2020 target for controlling trachoma [11]. Given the current surgical rate in Ethiopia, the country with the greatest burden of trichiasis, it will take more than 10 years to clear the backlog [12].

Many individuals with trichiasis, particularly those with mild disease, decline surgery, even when this is provided free and close to home [13–16]. Lack of time and fear of surgery are leading reasons for poor surgical uptake, suggesting a need for non-surgical, community-based management strategies for those declining surgery [8,13,17]. Poor surgical outcomes (recurrent trichiasis or an unsatisfactory cosmetic appearance) may also deter people from accepting surgery [5,18–20].

Epilation is a widespread traditional practice in many trachoma endemic societies, with up to 70% of people with trichiasis using this treatment strategy [3,6,13,21]. It involves the repeated plucking of lashes touching the eye with forceps [3,4,8]. Many individuals who decline surgical treatment consider epilation an acceptable alternative [13]. In view of the problems in delivering the necessary volume of surgery, the high rate of refusals in some areas and concerns about the quality of programmatic surgical outcomes, we conducted a randomized controlled non-inferiority trial of epilation versus surgery for minor trichiasis in Ethiopia; the two-year follow-up results have been reported [22]. With respect to the primary outcome of progression to major trichiasis there was an inconclusive result, relative to the predetermined non-inferiority margin of 10%. However, at two years there was no difference in the change in visual acuity or change in corneal disease between the two groups. At the two-year time-point all individuals who had been randomized at baseline to epilation were offered free surgery, however, only one third accepted. Here we report the four-year outcomes of study participants.

## Methods

### Ethics statement

This study was approved by the National Health Research Ethics Review Committee (NHRERC) of the Ethiopian Ministry of Science and Technology, the London School of Hygiene and Tropical Medicine Ethics Committee and Emory University Institutional Review Board. Potential participants were provided with both written and oral information in Amharic about the trial. For those agreeing to participate, written informed consent in Amharic was required prior to enrolment. If the participant was unable to read and write, the information sheet and consent form were read to them and their consent recoded by witnessed thumbprint. The detailed Trial Protocol is described in S1 Text and the CONSORT statement in Text S2 of the report of the two-year results [22].

### Trial summary: baseline to two-years

The trial methods and results up to two-years have been published previously [22]. Briefly, 1300 individuals aged 18 years or over with previously un-operated minor trichiasis were recruited in West Gojjam, Amhara Region, Ethiopia from March to June 2008. At baseline, unaided and pinhole LogMAR visual acuities were measured at 4 metres using an ETDRS equivalent Tumbling-E LogMAR chart and the eyes were examined using 2.5x magnification loupes by a single ophthalmologist (SR), and graded according to the detailed WHO FPC Trachoma grading system. Standardised high-resolution digital photographs were taken of each of the clinical features. In individuals with bilateral trichiasis one eye was randomly designated as the “study eye” although both eyes were treated. Following baseline assessment, participants were randomised to one of two intervention groups: (1) posterior lamella tarsal rotation surgery, or (2) repeated epilation using high quality, machine-manufactured epilation forceps (Tweezerman). Surgery was performed by five experienced Integrated Eye Care Workers (IECWs), chosen on the basis of the quality of their surgery. The surgeons received refresher training and underwent a standardisation process. Individuals randomised to the epilation group were each given two pairs of epilation forceps; the participant and an accompanying adult (“epilator”) with good near vision were trained to perform epilation. The procedure was explained and demonstrated to them by a field worker, who then in turn watched and checked the technique of the relative / patient in performing epilation.

Participants were followed-up at 6, 12, 18 and 24 months and re-assessed using the same protocol. Participants who showed evidence of disease progression during the follow-up period, defined as five or more lashes touching the eye or corneal changes related to observed lashes, were immediately offered primary surgery (epilation arm) or repeat surgery (surgery arm) to be performed by a senior surgeon. New epilating forceps were provided for epilation arm participants as required. Individuals with other ophthalmic pathology (e.g. cataract) were referred to the regional ophthalmic service in Bahirdar. At the end of the trial at two-years all epilation arm participants were offered free trichiasis surgery in the community. Some individuals accepted this, but the majority chose to continue epilating.

### Four-year follow-up assessment

About four years after enrolment participants were invited for a follow-up assessment (March to August 2012). They were notified by a letter sent out through the village administration teams, which explained the purpose, date and place of follow-up assessment. People not able to come to the health facilities for assessment were assessed in their homes. Reasons for loss to follow-up were identified and documented. Participants were interviewed in Amharic about their vision, ocular symptoms, epilation forceps retention and history of epilation and/or surgery since the two-year follow-up. Individuals were considered to be “frequent epilators” if they performed epilation at least once in two months. Participants enrolled into the epilation arm were asked about their views on epilation and epilation practices.

Unaided and pinhole LogMAR visual acuities were measured at 4 metres. Ophthalmic examinations were conducted in the same manner as the previous follow-ups by a single observer (EH) who had also conducted the 6 and 18-month follow-ups. Grades of trichiasis, entropion, and corneal opacity were documented and the eyes were photographed. The examiner was masked to the intervention allocation. The treatment allocation code had been previously broken for the two year analysis. Recurrent trichiasis was defined as one or more lashes touching the eye or evidence of epilation or repeat surgery. Clinical evidence of epilation was identified by the presence of broken or newly growing lashes, or areas of absent lashes. Change in corneal opacity was assessed by direct comparison of the baseline and four-year cornea photographs. Photographs were viewed on a computer screen at about 10x magnification by a single masked ophthalmologist (MJB). These were graded as improved, no change, or worse.

### Statistical analysis

Patients initially randomised to epilation were sub-divided according to whether or not they had surgery during the four-year follow-up period. These are subsequently referred to as epilation-to-surgery and epilation-only groups, respectively. Baseline and four-year follow-up demographic and clinical characteristics, and the change in clinical phenotypes during follow-up were compared between the surgery-only, epilation-only and epilation-to-surgery participants using X^2^ tests. The Wilcoxon rank-sum test was used to test for significant differences in number of lashes. Logistic regression was used to assess factors associated with trichiasis progression within the epilation-only group, to identify predictors of surgery uptake in all epilation arm participants and corneal opacity deterioration in all study participants. Ordinal logistic regression was used to assess factors associated with reduction in visual acuity by four-years in all study participants. Variables that were associated with the outcome on univariable analyses at a level of p<0.05 were retained in the multivariable logistic regression models.

## Results

### Participants and baseline characteristics

**Fig 1:**
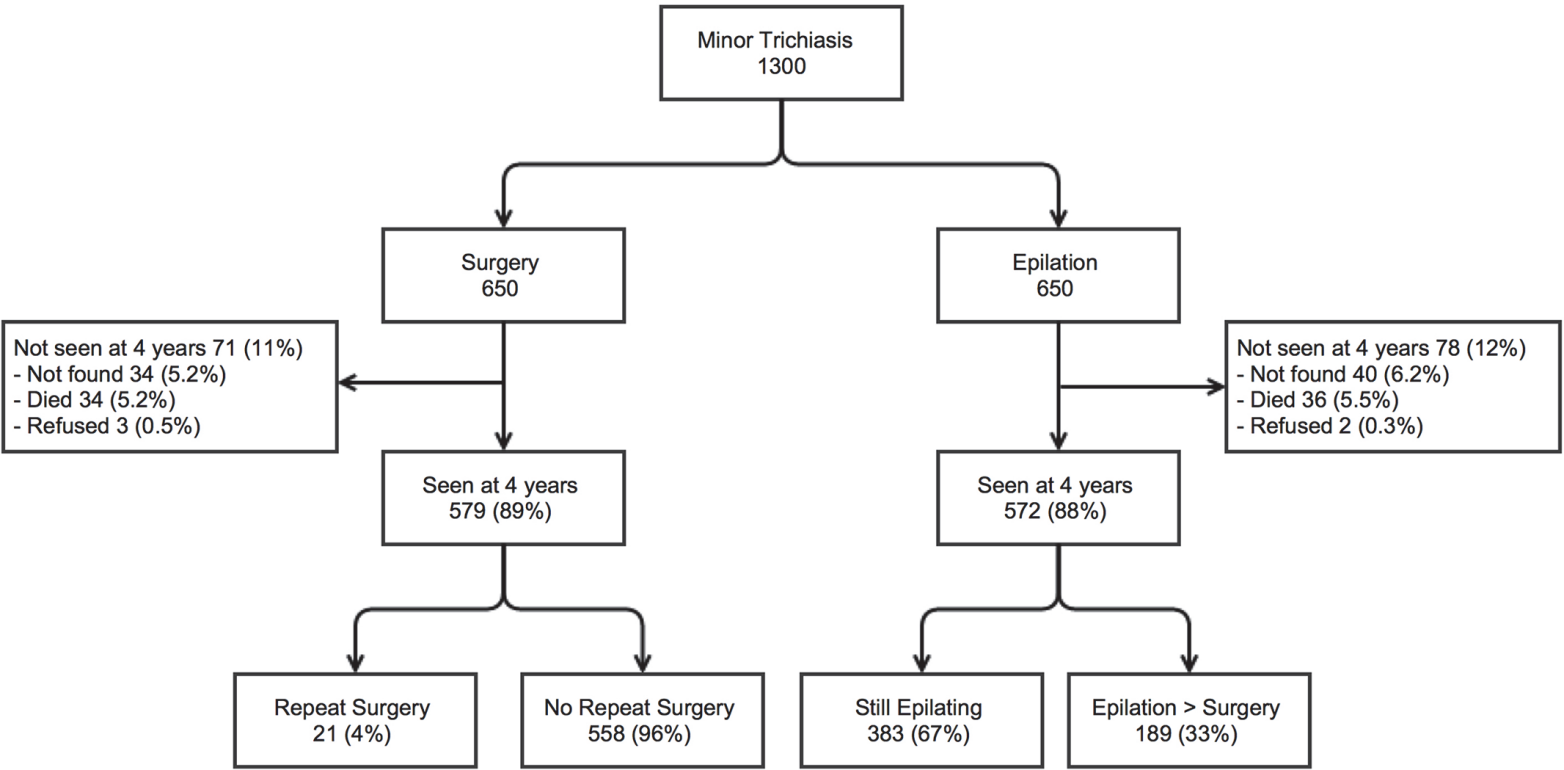
Study participant flow diagram.

At baseline, 1300 individuals were recruited, of whom 650 were randomised to immediate surgery and 650 to epilation (Fig. 1). The baseline demographic and clinical characteristics of all 1300 participants have been previously described along with the results up to two-years follow-up [22]. At four-years 1151 (88.5%) were re-examined: 579 (89%) participants from the surgery arm and 572 (88%) from the epilation arm. The baseline demographic and clinical characteristics of these individuals are shown in Table 1 (columns A and B). They were all Amharan with an average age of 50.3 years (SD 14.4; range 18–95) at baseline. The majority were female (767, 66.6%) and illiterate (1034, 89.8%). Of those initially randomised to the epilation arm, 189 (33%) had undergone trichiasis surgery by the four-years follow-up, while the other 383 (67%) were still epilating only (Fig. 1).

**Table 1:** Baseline demographic and clinical characteristics of participants seen at four years.

Characteristics		(A) Surgery Arm (579)		(B) Epilation Arm (572)		(C) Epilation-Only (383)		(D) Epilation-to-Surgery (189)		A v B	A v C	C v D
		*n*	*(%)*	*n*	*(%)*	*n*	*(%)*	*n*	*(%)*	*p-value*	*p-value*	*p-value*
**Gender. female**		403	(69.6%)	364	(63.6%)	243	(63.2%)	122	(64.6%)	0.032	0.038	0.750
**Age, mean years (SD)**		49.0	(14.0)	50.0	(13.9)	51.8	(13.5)	46.4	(14.1)	0.219	0.002	<0.001
**Illiterate**		517	(89.3%)	517	(90.4%)	344	(89.8%)	173	(91.5%)	0.540	0.795	0.512
**Lash Number**												
	Median (IQR)	1.0	(1–2)	1.0	(1–2)	1.0	(1–2)	1.0	(1–3)	0.376	0.709	0.008
**Trichiasis Distribution**												
	None (epilating)	106	(18.3%)	101	(17.7%)	67	(17.5%)	34	(18.0%)	0.632	0.481	<0.001
	1–2 lashes	360	(62.2%)	346	(60.5%)	251	(65.5%)	95	(50.3%)			
	3–4 lashes	94	(16.2%)	109	(19.1%)	58	(15.1%)	51	(27.0%)			
	5 lashes	19	(3.3%)	16	(2.8%)	7	(1.8%)	9	(4.8%)			
**Lash Location**												
	No lashes	106	(18.3%)	101	(17.7%)	67	(17.5%)	34	(18.0%)	0.564	0.064	0.005
	Corneal ± Peripheral	422	(72.9%)	410	(71.7%)	264	(68.9%)	146	(77.2%)			
	Peripheral	51	(8.8%)	61	(10.7%)	52	(13.6%)	9	(4.8%)			
**Entropion Grade**												
	0	244	(42.1%)	265	(46.3%)	200	(52.2%)	65	(34.4%)	0.340	0.020	0.001
	1	226	(39.0%)	216	(37.8%)	128	(33.4%)	88	(46.6%)			
	2	106	(18.3%)	90	(15.7%)	54	(14.1%)	36	(19.0%)			
	3	3	(0.5%)	1	(0.2%)	1	(0.3%)	0	-			
	4	0	-	0	-	0	-	0	-			
**Conjunctivalisation**												
	0	5	(0.9%)	5	(0.9%)	5	(1.3%)	0	-	0.455	0.311	0.288
	1	15	(2.6%)	22	(3.8%)	17	(4.4%)	5	(2.7%)			
	2	148	(25.7%)	129	(22.5%)	87	(22.6%)	42	(22.2%)			
	3	411	(70.9%)	416	(72.7%)	274	(72.7%)	142	(75.1%)			
**Visual Acuity**												
	-0.2–0.3	224	(38.9%)	192	(33.6%)	115	(30.0%)	77	(41.0%)	0.126	0.007	0.024
	0.3–0.7	245	(42.5%)	243	(42.6%)	166	(43.3%)	77	(41.0%)			
	0.7–1.1	66	(11.5%)	74	(13.0%)	54	(14.1%)	20	(10.6%)			
	1.1–2.0	15	(2.6%)	23	(4.0%)	15	(3.9%)	8	(4.3%)			
	CF/HM/PL/NPL	26	(4.5%)	39	(6.8%)	33	(8.6%)	6	(3.2%)			
	Not measurable^a^	3	-	1	-	0	-	1	-			
**Corneal opacity**												
	CC0	357	(61.7%)	311	(54.4%)	213	(55.6%)	98	(51.9%)	0.008	0.025	0.724
	CC1	127	(21.9%)	133	(23.3%)	84	(21.9%)	49	(25.9%)			
	CC2	85	(14.7%)	123	(21.5%)	83	(21.7%)	40	(21.2%)			
	CC3	10	(1.7%)	5	(0.9%)	3	(0.8%)	2	(1.1%)			

The participants were subdivided into the following groups: (A) Surgery Arm, (B) Epilation Arm, (C) Epilation-Only, and (D) Epilation-to-Surgery. The following comparisons were made: Surgery Arm to Epilation Arm (A v B), Surgery Arm to Epilation-Only (A v C) and Epilation-Only to Epilation-to-Surgery (C v D).

P-values were calculated by X^2^, with the exception of those for age and lash number differences, which were calculated by t-test and Wilcoxon rank-sum test, respectively.

CF, count fingers; HM, hand movements; PL, perception of light; NPL, no perception of light. CC0 to CC3: WHO Detailed Trachoma Grading System for corneal opacification.

^a^ Baseline VA was not measured in 2 people from those not seen at 4 years and 4 people from those seen at 4 years.

There were 149 participants who were not re-examined at four-years. The reasons for not being re-examined are shown in Fig. 1. In summary, at baseline this group was slightly older (p <0.001), had worse presenting LogMAR visual acuity (p <0.001) and had slightly more corneal opacification (p = 0.04) than the 1151 re-examined at four-years. Other variables such as sex, literacy and other baseline clinical characteristics including trichiasis severity and corneal opacity were comparable between those re-examined and not re-examined at four-years.

Amongst those re-examined at four-years, there were more female participants randomised to surgery compared to those randomisation to epilation (p = 0.03), however, there was no difference in age or literacy status (Table 1). Baseline clinical characteristics were balanced between the randomisation arms (Table 1), with the exception of central corneal opacity (CC2/CC3), which was more frequent in the epilation group (128; 22.4%) than the surgery group (95; 16.4%).

The 383 epilation arm participants who were still only epilating at four-years (epilation-only) were slightly older than both the 579 surgery arm and 189 epilation-to-surgery participants (mean ages 50.0, 49.0 and 46.4 years respectively; p<0.002, Table 1). Within the epilation arm, the epilation-only group had slightly less baseline trichiasis than the epilation-to-surgery group (p<0.001). The epilation-only group had slightly less baseline entropion than both the surgery arm (p = 0.02) and epilation-to-surgery participants (p = 0.001). The baseline LogMAR visual acuity of the epilation-only group was slightly worse than both the surgery-arm (p = 0.007) and the epilation-to-surgery arm (p = 0.02), Table 1.

### Trichiasis status at four-years

During the four-year period, recurrent trichiasis developed in 122 (21.1%) of the 579 participants randomised to surgery. Of these 122, 61 (50.0%) were practicing epilation at four years. Twenty-one (3.6%) had undergone repeat surgery during the four years. Among the 189 epilation-to-surgery participants, 42 (22.2%) had failed surgery (recurrent trichiasis), of whom 27 (64.2%) were epilating.

At four-years, among the 383 epilation-only participants, 207 (54.1%) were successfully epilating (had no lashes touching the eye), 166 (43.3%) had minor trichiasis (<6 lashes) and 10 (2.6%) had major trichiasis (>5 lashes). Overall, the epilation-only group had more trichiasis, entropion and lid margin conjunctivalisation than either the surgery arm or the epilation-to-surgery group (Table 2).

**Table 2:** Four-year clinical characteristics of participants.

Characteristics		(A) Surgery Arm (579)		(B) Epilation Arm (572)		(C) Epilation-Only (383)		(D) Epilation-to-Surgery (189)		A v C	C v D
		*n*	*(%)*	*n*	*(%)*	*n*	*(%)*	*n*	*(%)*	*p-value*	*p-value*
**Lash Number**											
	Median (IQR)	0	(0–0)	0	(0–1)	0	(0–2)	0	(0–0)	<0.001	<0.001
**Trichiasis Distribution**											
	None	548	(94.7%)	371	(64.9%)	207	(54.0%)	164	(86.8%)	<0.001	<0.001
	1–2 lashes	27	(4.7%)	133	(23.3%)	113	(29.5%)	20	(10.6%)		
	3–4 lashes	4	(0.7%)	41	(7.2%)	37	(9.7%)	4	(2.1%)		
	5+ lashes	0	-	27	(4.7%)	26	(6.8%)	1	(0.5%)		
**Lash Location**											
	No lashes	548	(94.7%)	371	(64.9%)	207	(54.0%)	164	(86.8%)	<0.001	<0.001
	Corneal±Peripheral	21	(3.6%)	185	(32.3%)	162	(42.3%)	23	(12.2%)		
	Peripheral	10	(1.7%)	16	(2.8%)	14	(3.7%)	2	(1.0%)		
**Entropion Grade**											
	0	559	(96.6%)	332	(58.0%)	159	(41.5%)	173	(91.5%)	<0.001	<0.001
	1	14	(2.4%)	144	(25.2%)	133	(34.7%)	11	(5.8%)		
	2	6	(1.0%)	92	(16.1%)	89	(22.7%)	5	(2.7%)		
	3	0	-	4	(0.7%)	4	(1.0%)	0	-		
	4	0	-	0	-	0	-	0	-		
**Conjunctivalisation**											
	0	192	(33.2%)	61	(10.7%)	16	(4.2%)	45	(23.9%)	<0.001	<0.001
	1	71	(12.3%)	25	(4.4%)	6	(1.6%)	19	(10.1%)		
	2	130	(22.5%)	53	(9.3%)	15	(3.9%)	38	(20.2%)		
	3	186	(32.1%)	432	(75.7%)	346	(90.3%)	86	(45.7%)		
**Visual Acuity**											
	-0.2–0.3	229	(39.6%)	200	(35.0%)	118	(30.9%)	82	(43.4%)	0.006	0.028
	0.3–0.7	223	(38.6%)	201	(35.2%)	142	(37.2%)	59	(31.2%)		
	0.7–1.1	49	(8.5%)	68	(11.9%)	52	(13.6%)	16	(8.5%)		
	1.1–2.0	13	(2.2%)	15	(2.6%)	12	(3.1%)	3	(1.6%)		
	CF/HM/PL/NPL	64	(11.1%)	87	(15.2%)	58	(15.2%)	29	(15.3%)		
	Not measurable	1	-	1	-	1	-	0	-		
**Corneal Opacity**											
	CC0	293	(51.1%)	273	(48.2%)	177	(46.8%)	96	(51.1%)	0.065	0.746
	CC1	203	(35.4%)	185	(32.7%)	125	(33.1%)	60	(31.9%)		
	CC2	71	(12.4%)	100	(17.7%)	70	(18.5%)	30	(16.0%)		
	CC3	7	(1.2%)	8	(1.4%)	6	(1.6%)	2	(1.1%)		

The participants were subdivided into the following groups: (A) Surgery Arm, (B) Epilation Arm, (C) Epilation-Only and (D) Epilation-to-Surgery. The following comparisons were made: Surgery Arm to Epilation-Only (A v C) and Epilation-Only to Epilation-to-Surgery (C v D).

P-values were calculated by X^2^, with the exception of those for the lash number differences, which were calculated by the Wilcoxon rank-sum test. CF, count fingers; HM, hand movements; PL, perception of light; NPL, no perception of light. CC0 to CC3: WHO Detailed Trachoma Grading System for corneal opacification.

Changes in clinical phenotype between baseline and four-years are shown in Table 3. The outcomes of surgery in terms of trichiasis, entropion and conjunctivalisation were mostly very good. In the epilation-only group, the number of lashes touching the eye increased in 82 (21.5%), however, this was mostly a minor increase, with progression from minor to major trichiasis in six (1.6%) of the 383 epilation only patients. The majority of individuals had less or the same level of trichiasis. Independent risk factors for 5+ lashes touching were baseline age ≥50 years, ≥3 lashes at baseline, and infrequent epilation (Table 4).

**Table 3:** Change in clinical phenotype between baseline and four-years.

Characteristics		(A) Surgery Arm (579)		(B) Epilation Arm (572)		(C) Epilation-Only (383)		(D) Epilation-to-Surgery (189)		A v B	A v C	C v D
		n	(%)	n	(%)	n	(%)	n	(%)	*p-value*	*p-value*	*p-value*
**Trichiasis**												
	≥5 lashes more	0	-	6	(1.1%)	6	(1.6%)	0	-	<0.001	<0.001	<0.001
	3–4 lashes more	0	-	21	(3.7%)	21	(5.5%)	0	-			
	1–2 lashes more	5	(0.9%)	63	(11.0%)	55	(14.4%)	8	(4.2%)			
	No change	120	(20.7%)	138	(24.1%)	101	(26.4%)	37	(19.6%)			
	1–2 lashes less	346	(59.8%)	262	(45.8%)	172	(44.9%)	90	(47.6%)			
	3–4 lashes less	92	(15.9%)	73	(12.8%)	25	(6.5%)	48	(25.4%)			
	≥5 lashes less	16	(2.8%)	9	(1.6%)	3	(0.8%)	6	(3.2%)			
**Entropion**												
	>1 grade worse	5	(0.9%)	32	(5.6%)	31	(8.1%)	1	(0.5%)	<0.001	<0.001	<0.001
	1 grade worse	2	(0.3%)	116	(2.3%)	109	(28.5%)	7	(3.7%)			
	No change	243	(42.0%)	234	(40.9%)	166	(43.3%)	68	(36.0%)			
	1 grade better	227	(39.2%)	140	(24.5%)	62	(16.2%)	78	(41.3%)			
	>1grade better	102	(17.6%)	50	(8.7%)	15	(3.9%)	35	(18.5%)			
**Conjunctivalisation**												
	>1 grade worse	5	(0.9%)	10	(1.8%)	10	(2.6%)	0	-	<0.001	<0.001	<0.001
	1 grade worse	26	(4.5%)	93	(16.3%)	79	(20.6%)	14	(7.4%)			
	No change	190	(32.8%)	356	(62.3%)	271	(70.8%)	85	(45.2%)			
	1 grade better	131	(22.6%)	46	(8.1%)	12	(3.1%)	34	(18.1%)			
	>1 grade better	227	(39.2%)	66	(11.7%)	11	(2.9%)	55	(29.3%)			
**Visual Acuity** ^**a**^												
	>-0.3 much worse	83	(14.4%)	102	(17.9%)	63	(16.5%)	39	(20.9%)	0.271	0.892	0.219
	-0.1 to -0.3 worse	109	(19.0%)	118	(20.7%)	75	(19.6%)	43	(23.0%)			
	Within 0.1 (same)	240	(41.7%)	205	(36.0%)	150	(39.2%)	55	(29.4%)			
	0.1 to 0.3 better	98	(17.0%)	102	(17.9%)	66	(17.5%)	36	(18.7%)			
	>0.3 much better	45	(7.8%)	43	(7.5%)	28	(7.3%)	15	(8.0%)			
**Corneal Opacification** ^**b**^												
	More opacity	8	(1.4%)	13	(2.3%)	9	(2.4%)	4	(2.1%)	0.467	0.377	0.668
	No Change	558	(97.2%)	548	(96.7%)	367	(96.8%)	181	(96.3%)			
	Less opacity	8	(1.4%)	6	(1.1%)	3	(0.8%)	3	(1.6%)			

The clinical assessment included a change in trichiasis, entropion, conjunctivalisation, visual acuity and corneal opacification. The following comparisons were made: Surgery Arm to Epilation Arm (A v B), Surgery Arm to Epilation-Only (A v C) and Epilation-Only to Epilation-to-Surgery (C v D).

P-values were calculated by X^2^.

^a^ Four individuals in the surgery arm and two individuals in the epilation arm did not have visual acuity measured at both baseline and four years.

^b^ Ten individuals did not have paired corneal photographs from both baseline and four years.

**Table 4:** Univariable and multivariable associations with the presence of 5+ lashes in Epilation-Only patients at four-years.

Variable	OR	95% CI	p-value
**Univariable analysis**			
Age, ≥50 years	3.04	(1.12–8.25)	0.029
Gender, Female	1.63	(0.67–3.98)	0.283
Baseline lash number ≥ 3 lashes	3.43	(1.48–7.96)	0.004
Entropion progression	1.09	(0.48–2.48)	0.834
Not Epilating frequently at 4 years	2.65	(1.04–6.75)	0.042
**Multivariable logistic regression model**			
Age, ≥50 years	2.97	(1.09–8.18)	0.035
Baseline lash number ≥ 3 lashes	3.51	(1.49–8.29)	0.004
Not Epilating frequently at 4 years	2.54	(0.98–6.59)	0.054

### Visual acuity at four-years

At four-years the surgery arm and epilation-to-surgery participants had slightly better LogMAR visual acuity than the epilation-only group (Table 2). However, this difference is attributable to the pre-existing difference in baseline vision (reported above, Table 1), as there was no difference between the different groups in terms of change in visual acuity between baseline and four-years (Table 3). Age ≥50 years, male gender, detection of other visually impairing conditions (e.g. cataract), baseline corneal opacification (CC2/CC3) and incident/progressive corneal opacification were independently associated with deterioration in visual acuity (Table 5).

**Table 5:** Univariable and multivariable associations with visual acuity deterioration by four-years, amongst all individuals seen at four years (surgery and epilation arms).

Variable	OR	95% CI	p-value
**Univariable analysis**			
Age, ≥50 years	1.81	(1.47–2.24)	<0.001
Sex, Female	0.59	(0.47–0.74)	<0.001
Treatment Arm, Epilation	1.14	(0.93–1.41)	0.217
Lash number at 4 years ≥ 3 lashes	1.33	(0.86–2.06)	0.207
Other visually impairing conditions identified*	5.41	(2.71–10.8)	<0.001
Baseline corneal opacity (CC2/CC3)	1.68	(1.27–2.23)	<0.001
Incident or progressive corneal opacity at 4 years	3.60	(1.50–8.64)	0.004
**Multivariable ordinal logistic regression**			
Age, ≥50 years	1.45	(1.16–1.82)	0.001
Sex, Female	0.67	(0.53–0.85)	0.001
Other visually impairing conditions identified*	4.68	(2.27–9.64)	<0.001
Baseline corneal opacity (CC2/CC3)	1.40	(1.05–1.87)	0.021
Incident or progressive corneal opacity at 4 years	2.70	(1.11–6.60)	0.029

* 25 people had other visually impairing conditions identified: 20 cataract, 2 glaucoma, 1 Aphakic, 1 Evisceration, 1 corneal ulcer

### Corneal opacification at four-years

Overall, few individuals had a change in corneal opacification, determined by the comparison of baseline and four-year photographs (Table 3). There was no difference in change of corneal opacification between the surgery arm and the epilation-only group or the epilation-to-surgery group (Table 3). Incident or progressive corneal opacification was independently associated with age ≥50 years and the presence of some baseline corneal opacification (CC2/CC3), Table 6.

**Table 6:** Univariable and multivariable associations with corneal opacity deterioration (incident and progressive) by four-years, amongst all individuals seen at four years (surgery and epilation arms).

Variable	OR	95% CI	p-value
**Univariable analysis**			
Age, ≥50 years	4.43	(1.29–15.1)	0.017
Sex, Female	0.45	(0.19–1.07)	0.069
Treatment Arm, Epilation	1.66	(0.68–4.04)	0.263
Lash number at 4 years ≥ 3 lashes	0.74	(0.10–5.58)	0.769
Baseline corneal opacity (CC2/CC3)	3.19	(1.33–7.68)	0.009
**Multivariable logistic regression**			
Age, ≥50 years	3.85	(1.11–13.3)	0.033
Baseline corneal opacity (CC2/CC3)	2.67	(1.10–6.47)	0.030

### Epilation practice at four-years

Among the epilation-only group 259 (67.6%) were “frequent epilators” (at least once in two months) between the two and four-year follow-ups. They were asked about their experience: 185 (72%) reported “no problem”, 37 (14.3%) did not always find the trained epilators when needed, 17 (6.6%) had found epilation uncomfortable, the trained epilators of 9 (3.5%) reported difficulty epilating, and 7 (2.7%) had found people unwilling to epilate them. Among the 124 who were not frequently epilating, 119 (96%) did not have a specific reason for not epilating other than not needing to; the other five had nobody to perform epilation. Epilation frequency was not associated either with age (p = 0.31) or gender (p = 0.60). Compared to the “infrequent epilators”, the “frequent epilators” had a slightly higher lash burden at baseline (Median: 1 vs 1, Wilcoxon rank-sum test, p = 0.19), but lower lash burden at four-years (Median: 0 vs 1, Wilcoxon rank-sum test, p = 0.073).

The epilation-only group were asked if they had tried to obtain trichiasis surgery at any time between the two and four-year follow-up: 352 (92%) replied “Never” and 325 (85%) reported that they were happy epilating. There was no statistically significant difference in the average lash burden at four-years between those who were happy epilating and those that were not (1.11 v 1.31, p = 0.30). Participants who were not happy epilating were more likely to have tried to obtain surgical treatment for their trichiasis between two and four-year follow-ups (Fisher’s exact test, p = <0.001).

At the two-year follow-up, 589 / 603 (98%) of epilation arm participants still had their epilation forceps. At the four-year follow-up, 351 / 383 (92%) of the epilation-only group had retained at least one pair of epilation forceps. Females were more likely to have retained their forceps than males (OR 2.38; 95%CI 1.15–4.96; p = 0.020). At four-years, new forceps were provided to those who had lost their forceps, and did not want surgery.

### Surgery uptake by the epilation arm participants

Univariate and multivariable associations with having surgery in epilation arm participants at any point during the four years of follow-up are shown in Table 7. Having surgery was independently associated with age less than 50 years, ≥3 lashes or corneal lashes at baseline, and frequent baseline epilation.

**Table 7:** Univariable and multivariable associations with accepting trichiasis surgery by four-years in all participants randomised to the epilation arm.

Variable	OR	95% CI	p-value
**Univariable analysis**			
Age, ≥50 years	0.53	(0.37–0.75)	<0.001
Sex, Female	1.06	(0.74–1.53)	0.750
Baseline lash number ≥ 3 lashes	2.27	(1.52–3.42)	<0.001
Baseline lash location (Corneal lashes)	3.19	(1.53–6.67)	0.002
Epilating frequently at baseline	2.32	(1.60–3.38)	<0.001
**Multivariable logistic regression**			
Age (≥50 years)	0.52	(0.34–0.78)	0.001
Baseline lash number ≥ 3 lashes	2.17	(1.40–3.39)	<0.001
Baseline lash location (Corneal lashes)	2.83	(1.33–6.05)	0.006
Epilating frequently at baseline	2.39	(1.57–3.63)	<0.001

### Management at four-years

At the four-year follow-up, all participants with recurrent trichiasis in the surgery arm and all participants in the epilation arm who had not previously had surgery were offered free surgery: only 17 / 383 (4.4%) of the epilation only participants accepted surgery, the remaining 366 (95.6%) preferred to continue epilating.

## Discussion

Trachomatous trichiasis has a wide disease spectrum, with many individuals having relatively few lashes touching the eye [4,7]. This may partly explain the observation in this study that at two-years, despite being offered surgery free of charge and close to home, more than two-thirds of people practicing epilation declined surgery. Most (92%) of the epilation-only patients had not sought trichiasis surgery during the two to four-year follow-up period, and the majority (85%) reported that they were still happy epilating. This was also reflected in 96% of the epilation-only patients declining the offer of free community-based surgery at the time of the four-year follow-up. This finding is consistent with two Gambian cohort studies, in which 50–70% of individuals with major trichiasis declined trichiasis surgery [8,13]. Presence of symptoms interfering with work was a predictor for accepting surgery [13]. In our study, younger patients and those with higher baseline lash burden, corneal lashes and frequent epilation at baseline were more likely to accept surgery. It seems likely that these individuals are more symptomatic and therefore more motivated to find a potential long-term solution in surgery. It is encouraging to note that patients with corneal lashes and higher lash burden are more willing to accept surgical management, as these are strong indications for surgery.

In this study, surgery was better than epilation at correcting entropion and controlling trichiasis. However, it should be noted that at four-years 76.2% of the epilation-only participants had mild or no entropion, and 63.4% had no entropion progression. The epilation-only group generally controlled their trichiasis well by epilation, with only a few showing signs of significant progression. At four-years 2.6% of this group had major trichiasis (>5 lashes), which is low compared to the Gambian longitudinal study, in which 37% of the eyes progressed from minor to major trichiasis over four years [8]. However, in the Gambian study participants used low quality traditional epilation forceps without training. In our original trial analysis with follow-up to two-years, the primary endpoint was the presence of 5+ lashes touching or a history of surgery. At four-years only 6.8% of the epilation only group had 5+ lashes. This is somewhat less that the cumulative total of 13.9% individuals who had reached the primary endpoint by two-years, many of whom had accepted surgery at two years.

The proportion of participants in the surgery arm with recurrent trichiasis at four-years was relatively low compared to other trials and longitudinal studies [18,23,24]. This is because the risk of recurrence is heavily influenced by pre-operative disease severity; all the participants in our study had minor trichiasis at baseline; other studies have enrolled patients with more severe disease [5,22,25,26].

The epilation-only group had poorer baseline visual acuity compared to both the surgical arm participants and the epilation-to-surgery group. However, there was no difference in visual acuity change (baseline to four-years) between the epilating and surgery groups, which is similar to what we reported at two-years [22]. Several studies have reported an overall improvement in visual acuity after trichiasis surgery [5,27]. However, participants in these studies, unlike those in our present study, had a wider range of baseline trichiasis severity and were therefore more likely to have an improvement in vision following trichiasis surgery. Consistent with other studies, older age, presence of other blinding conditions, baseline corneal opacity and progressive corneal opacification were associated with deterioration of vision [8]. It is likely that much of the reduction in visual acuity over the four years is due to age related changes such as cataract. Interestingly, we found that female participants had 33% less risk of loss of vision than males. The explanation for this is unknown. The proportion of participants with visual impairment and blindness increased markedly at four-years in all groups, pointing to a major burden of blindness in the study area from other causes such as cataract in this older group of people.

The epilation arm as a whole and the epilation-only sub-group had more baseline corneal opacity than the surgery arm participants. However, there was no significant difference in change of corneal opacity between the different groups at four-years. This result is consistent with our findings at two-years and a report from a longitudinal study in The Gambia, which compared change in corneal opacity in individuals with minor trichiasis who had undergone surgery with those who had declined surgery and practiced epilation [8]. In farming communities, corneal opacity can occur from other causes such as corneal infections and injuries. Similarly, new corneal opacity has been reported after surgery without the presence of recurrent trichiasis [18]. Corneal opacity development or progression was associated with old age and the presence of some pre-existing opacification, similar to other studies [5,6].

More than two-thirds of the epilation-only participants reported frequent epilation. Most reported no problems. Difficulty of getting the trained epilator when they needed help was cited as the main problem, encountered by 14%. However, this could be addressed by training more than one family member. The high retention rate of forceps in this study is encouraging, suggesting that the forceps are valued.

This study has a number of limitations. This four-year follow-up and analysis was not pre-specified in the original trial protocol, which covered the period up to the two-year follow-up. The study ceased to have a fully randomised treatment allocation at two-years when all the epilation arm participants were offered free surgery, and hence we have adjusted follow-up outcomes for baseline imbalances. Those not examined at four-years were older and had worse baseline presenting visual acuity than those seen at four-years. This could have underestimated change in vision over time as older age is associated with greater visual impairment. However, this is unlikely to introduce bias in vision change between the surgery arm and epilation only group as those lost to follow-up were equally distributed between these groups.

We found that surgery was more effective for controlling trichiasis than epilation; however there was no difference in change in visual acuity and corneal opacity. The progression of minor trichiasis can be effectively mitigated with frequent epilation. We found low rates of surgery uptake among people with mild disease, even with free community-based surgery. There is a need for clear guidelines on how programmes should manage patients with a few non-entropic lashes who refuse surgery. Trichiasis in general and particularly major trichiasis warrants surgical treatment. However, the results of this study and the reality of low surgical uptake in many regions, suggest that good quality epilation, in the context of regular follow-up by a service that can provide surgery if subsequently needed, is a reasonable second-line alternative to surgery for minor trichiasis for individuals who either decline surgery or do not have immediate access to surgical treatment.
